# Multiscale Aggregate Networks with Dense Connections for Crowd Counting

**DOI:** 10.1155/2021/9996232

**Published:** 2021-11-11

**Authors:** Pengfei Li, Min Zhang, Jian Wan, Ming Jiang

**Affiliations:** Hangzhou Dianzi University, Baiyang Road No. 2, Hangzhou, China

## Abstract

The most advanced method for crowd counting uses a fully convolutional network that extracts image features and then generates a crowd density map. However, this process often encounters multiscale and contextual loss problems. To address these problems, we propose a multiscale aggregation network (MANet) that includes a feature extraction encoder (FEE) and a density map decoder (DMD). The FEE uses a cascaded scale pyramid network to extract multiscale features and obtains contextual features through dense connections. The DMD uses deconvolution and fusion operations to generate features containing detailed information. These features can be further converted into high-quality density maps to accurately calculate the number of people in a crowd. An empirical comparison using four mainstream datasets (ShanghaiTech, WorldExpo'10, UCF_CC_50, and SmartCity) shows that the proposed method is more effective in terms of the mean absolute error and mean squared error. The source code is available at https://github.com/lpfworld/MANet.

## 1. Introduction

**Figure 1:**
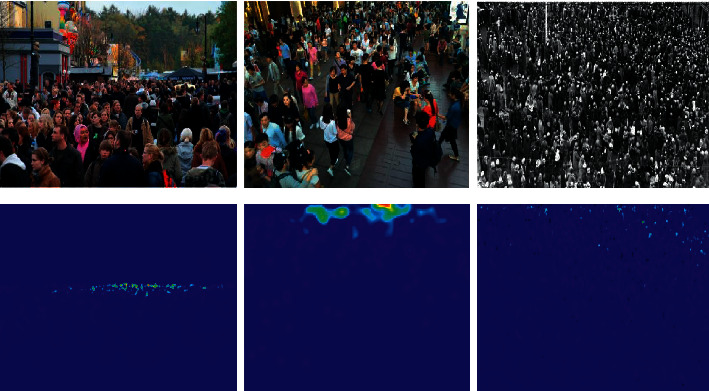
Images and ground truth density maps using the ShanghaiTech dataset [11].

Crowd counting technology is widely used in video surveillance, crowd management, traffic control, and other fields as well as at sporting events and political meetings [1, 2]. Crowd counting methods can also be extended to indirectly related fields, such as medical image analysis and animal group behavioral analysis [3]. Although the relevant research has achieved good results, considerable challenges persist owing to large-scale variations, heavy occlusion, background noise, and perspective distortion (Figure 1).

Researchers have proposed different approaches to solve these problems. For example, numerous multicolumn networks have been proposed. Multicolumn architectures involve several columns of a convolutional neural network (CNN) with different receptive fields to accommodate multiscale crowds [4–7]. Although these methods have achieved good results, the multicolumn structure induces a considerable increase in parameters and computational costs. Furthermore, the similarity of column networks results in a high redundancy of learning features [8–10]. The goal of our architecture is to retain more multiscale contextual features. The proposed network comprises an encoder that can extract and retain the required features and a decoder that gradually recovers the image resolution and interprets the encoded features.

A feature contains different information at different layers of the neural network. Most crowd counting methods use a 1 × 1 convolution to transform the feature of the last layer of the network into a density map. However, these methods ignore the relation between different layer features. We use dense connections to improve the structure and integrate the features of different layers.

Dilated convolution can effectively expand the receiving field without increasing the number of parameters and computational costs [11–13]. Li et al. [8] proposed a congested scene recognition network (CSRNet) by combining VGG-16 and dilated convolution layers to aggregate multiscale contextual features. Chen et al. [14] proposed a scale pyramid network, which contains different dilated convolution rates in parallel for multiscale information extraction. Although these methods show good performance in many tasks, the design of dilated convolution modules usually has excessive memory size requirements. Therefore, the modules must consider efficiency and effectiveness through processing operations.

**Figure 2:**
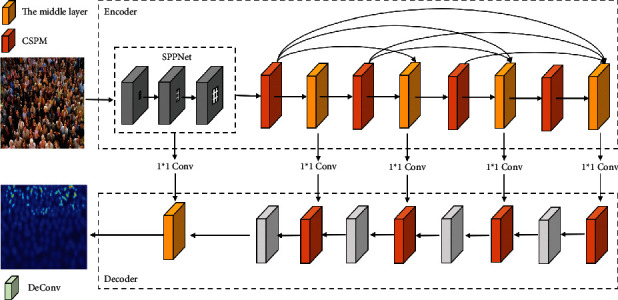
Architecture of the proposed MANet for crowd counting.

In this study, we propose a multiscale aggregation network (MANet) for crowd counting (Figure 2). The proposed MANet is an encoder-decoder network that uses a densely connected multiscale aggregation module in the encoder, referred to as a cascade scale pyramid network (CSPN). The CSPN contains four parallel dilated convolutions with different dilated rates for capturing the features of different receptive fields. The features obtained using the four dilated convolutions are further fused in a cascade manner to improve the ability of the network to handle multiscale features and anti-interference. Furthermore, the dimension reduction operation reduces redundant computations that are typical of deep convolution networks. To restore the resolution of the features, we use deconvolutions with different parameters to act on the features of different layers in the decoder. The loss function contains a Euclidean loss and a mean squared error loss, which form a valid training loss function. We conduct experiments using four major datasets (ShanghaiTech [7], SmartCity [9], WorldExpo'10 [15], and UCF_CC_50 [16]), achieving excellent results.

## 2. Related Work

A series of excellent crowd counting methods have been proposed [1, 17]. These methods can be categorized as detection-based, regression-based, and CNN-based approaches.

### 2.1. Detection-Based Approaches

Earlier, detection-based methods used a sliding window for target detection, including the manual extraction of the features of the human body or specific parts [18], such as the Haar wavelet [19] and histogram of oriented gradients [20]. To improve detection accuracy, researchers have analyzed crowd scenes by detecting specific body parts rather than the entire body [21]. Recently, researchers have attempted to employ CNN-based object detectors to count objects, such as YOLO [22], SSD [23], and faster RCNN [24]. However, even if only a pedestrian's head or smaller body parts are detected, these methods often cannot handle high-density crowd scenes owing to occlusion and illumination in crowded scenes.

### 2.2. Regression-Based Approaches

Regression-based approaches for crowd counting cannot accurately locate pedestrians. However, they can provide more accurate count estimates in crowded scenes. In particular, the regression-based approaches include feature-based regression approaches and density estimation-based regression approaches.

#### 2.2.1. Feature-Based Regression Approaches

Feature-based regression approaches attempt to extract various features from local image blocks [25–27]. Foreground or textural features are used to generate low-level information. Similar methods have been formulated based on Fourier analysis, SIFT [28], and interest points [29]. Feature-based regression methods handle occlusion and clutter effectively. However, they ignore scale information.

#### 2.2.2. Density Estimation-Based Regression Approaches

Density estimation-based regression methods consider the relation between image features and data regression. Lempitsky and Zisserman [30] proposed a linear mapping method considering local region features and density maps. Pham et al. [31] attempted to use random forest regression to realize a nonlinear map. Based on these studies, many density estimation-based regression methods for crowd counting have been developed [17, 32, 33].

### 2.3. CNN-Based Approaches

CNN-based approaches have achieved good results in crowd counting. A detailed CNN-based counting survey can be found in the literature [17]. Sam et al. [4] adopted a density classifier to classify image patches into appropriate CNN columns as inputs. A previous study proposed a CP-CNN [6] that involves two-column networks to extract both global and local contextual information. The network maps the input data to a high-dimensional feature map and then inputs the previously extracted contextual information set into the final fusion network to obtain a high-quality density map. In SAANet [34], global and local attention weights are used to capture variations in the crowd density between and within images. This proposed attention mechanism for network usage can automatically focus on local and global scales. SANet [10] attempted to extract multiscale head information from each image using a similar front-end network module. Furthermore, the final density map is obtained by deconvolution using different-sized convolution kernels in each layer. Although these CNN-based methods show good crowd counting ability, they have several disadvantages. These networks with redundant parameters and slow convergence are difficult to train to solve the problems of multiscale and occlusion.

Some studies have proposed crowd counting methods. DecideNet [35] used detection-based methods to count crowds in sparse crowd scenes and regression-based methods to count crowds in dense scenes and adopted an attention mechanism to regulate the use of the two methods. Sam et al. [36] proposed locating each person in a dense crowd using a bounding box to size the identified heads and then counting them. Another study proposed an adaptive dilated convolution that can learn a continuous hole rate at different positions in the image to effectively match changes in the scale at different positions [37]. PACNN [38] framework eliminates the need for a density regression paradigm. The specific operation involves encoding the input as perspective perception layers and adaptively combining multiscale density maps. Using ASNet [39], intermediate-density maps and scaling factors are first generated and then multiplied by the attention mask to output multiple density maps at different density levels based on the attentional mechanism. The final density map is obtained by combining these density maps.

## 3. Proposed Approach

An overview of the proposed model is shown in Figure 2. In this section, we describe the proposed model. In Section 3.1, we introduce the cascaded scale pyramid network (CSPN). In Sections 3.2 and 3.3, we describe the feature extraction encoder (FEE) and density map decoder (DMD), respectively. Network parameters are introduced in Section 3.4.

### 3.1. Cascaded Scale Pyramid Network (CSPN)

**Figure 3:**
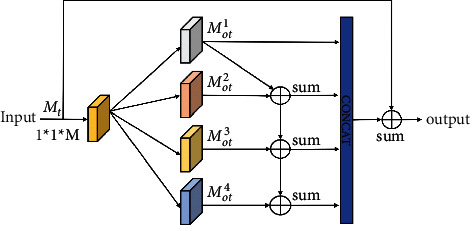
Computational process of the cascaded scale pyramid network.

The scale often varies continuously across the image and shows a large range. A network structure that achieves better results usually contains more complex designs. Considering these challenges, we propose a CSPN, which can balance efficiency and effectiveness. The standard convolution can be divided into two steps [40]. In the first step, pointwise convolution is used to reduce the dimension. In the second step, multiscale features are extracted using the spatial pyramid of dilated convolution. Motivated by this idea, we define the computational process of our module (Figure 3).

First, an *M*-dimensional input is reduced to a *d*-dimensional input using *d* convolution kernels of 1 × 1 × *M*. Then, four dilated convolutions with different dilated rates are used to parallel compute the feature output from the previous step; subsequently, four features of the same size are obtained. Finally, these four features are cascaded, and the result is added to the original input features to obtain the final output.

(1) Pointwise convolution converts high-dimensional features into low-dimensional features, realizing the fusion of cross-channel information and increasing the nonlinearity of the network
(2) The low-dimensional features are calculated using the parallel dilated convolution with different dilated rates (*d*1 = 1, *d*2 = 4, *d*3 = 8, and *d*4 = 16), which can rapidly increase and capture multiple receptive field information. Each dilated convolution in the CSPN possesses the same number of channels. For a given feature, the size of the receptive field is 3 × 3, 9 × 9, 17 × 17, and 33 × 33 for the features extracted using four dilated convolutions
(3) The outputs are fused to eliminate the “gridding issue,” and the output of the scale pyramid is obtained as follows:
  (1) $$\begin{array}{c} M_{ot}^{\left(s+1\right)}=M_{ot}^{s}+M_{ot}^{\left(s+1\right)},\;S>1, \end{array}$$
where *M*_*ot*_^*s*^ represents the fused features of the *s*-th layer. The features are spliced together to obtain the output of the scale pyramid *M*_*ot*_ ∈ *R*^*H∗W∗*∑_*s*=1_^4^*C*_*s*_^, where W and H represent the width and height of the feature map, respectively, and *C*_*s*_ represents the number of output channels for different columns.

### 3.2. Feature Extraction Encoder (FEE)

**Figure 4:**
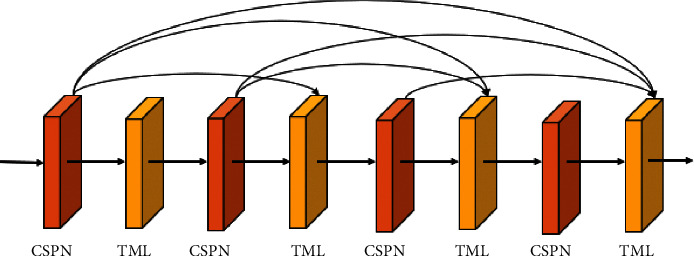
Dense connections in cross-layer connections.

We employ SPPNet as the front-end network of the encoder and input the generated feature to the CSPN. Four CSPNs, which are connected using specific rules, are used (Figure 4). The current CSPN improves information flow within the underlying network by sharing the features of the previous CSPN.

If the dense connection method is adopted and each layer produces *k* features, *k*_0_+*k*(*i* − 1) features will be input to the *i*-th layer. Here, *k*_0_ is the number of channels in the input layer and the hyperparameter *k* is the growth rate of the network. A larger *k* value signifies that the amount of information that flows in the network increases, the ability to extract features becomes stronger, and the number of model calculations increases.

Since each layer of the network will receive the features of all previous layers as inputs, there is a middle layer behind each densely connected block for dimensionality reduction. We set the compression factor *θ*(0 < *θ* ≤ 1) for dense connections. When *θ*=1, the channel number of output features does not change. In the middle layer, all *θ* values are considered to be 0.5, implying that the middle layer reduces the number of output channels to half the number of inputs.

### 3.3. Density Map Decoder (DMD)

CNN-based methods generate a low-resolution density map during continuous convolution and pooling, owing to which details of the crowd are usually lost [10, 14]. We use four fusion layers to progressively refine the details of the features to obtain a high-quality density map. Four deconvolutions are used to restore the image map resolution. When using deconvolution operations, the number of input channels is the same as the number of output channels. Finally, we adopt a 1 × 1 convolution to generate a high-resolution density map, which has the same resolution as the input image.

### 3.4. Loss Function

The Euclidean distance is used to assess the difference between the training density map and the model output density map. Based on this assessment, model parameters are adjusted to produce a density map that closely depicts the ground truth. The Euclidean loss function can provide an estimation error at the pixel level. The loss function is expressed as follows:

(2) $$\begin{array}{c} L_{E}=\frac{1}{2N}\sum_{i=1}^{N}{\left\|F\left(X_{i};\theta \right)-F_{i}\right\|}_{2}^{2}, \end{array}$$

where *F*(*X*_*i*_; *θ*) denotes the output of MANet, *θ* represents the variable model parameters, *X*_*i*_ denotes the input image, and *F*_*i*_ represents the ground truth result.

In addition, the mean absolute error (MAE) loss function is introduced to determine the count and estimated values as follows:

(3) $$\begin{array}{c} L_{c}=\frac{1}{N}\sum_{i=1}^{N}{\left|C\left(I_{i}\right)-C^{\prime }\left(I_{i}\right)\right|}_{2}^{2}, \end{array}$$

where *I*_*i*_ represents the density map generated using MANet, *C*(*I*_*i*_) represents the estimated count, and *C*′(*I*_*i*_) denotes the label value. To weigh the loss, the final loss function is expressed as follows:

(4) $$\begin{array}{c} \text{Loss}=L_{E}+\alpha L_{c}, \end{array}$$

where *α* is the super weight parameter, which was set to 0.01.

## 4. Experiments

**Figure 5:**
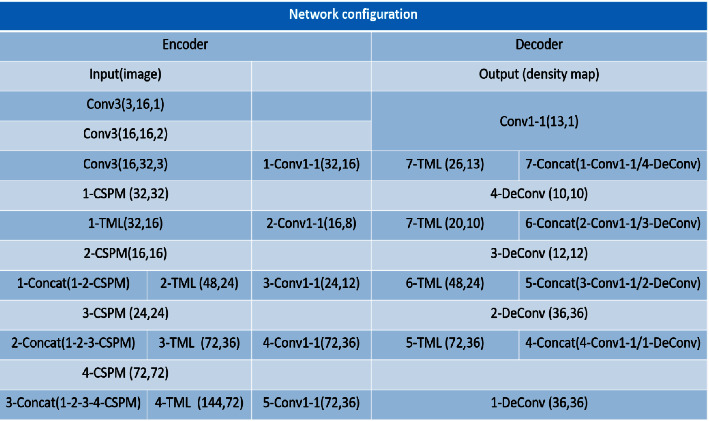
Network configuration. Convolution layer parameters are described as Conv (kernel size)_(number of filters)_(dilated rate), except Conv1-1 without the dilated rate. TML represents the middle layer. We assign a sequence number to identify each module. For example, 1-Concat (1-2 CSPN) represents the connection between 1-CSPN and 2-CSPN. 2-TML (48, 24) represents the second TML module with an input/output channel count of 48 and 24.

We evaluate the proposed MANet using four datasets (ShanghaiTech [7], SmartCity [9], WorldExpo'10 [15], and UCF_CC_50 [16]). First, we introduce the evaluation metrics, ground truth generation, and training details. Then, we compare the proposed method with state-of-the-art methods using these datasets. Finally, we demonstrate the effectiveness of our module via ablation experiments. The experiments were implemented in Pytorch, and the detailed network configuration is shown (Figure 5).

### 4.1. Evaluation Metrics

Based on the existing literature, the evaluation metrics are the MAE and mean squared error (MSE), which can be used to evaluate the performance of crowd counting methods. The MAE indicates the accuracy of the count, and the MSE represents the robustness of the model. The MAE and MSE are calculated as follows:

(5) $$\begin{array}{c} \text{MAE}=\frac{1}{N}\left|Z_{i}-Z_{i}^{\prime }\right|,\\ \text{MSE}=\sqrt{\frac{1}{N}\sum_{i=1}^{N}{\left(Z_{i}-Z_{i}^{\prime }\right)}^{2}}, \end{array}$$

where *N* represents the number of test images, *Z*_*i* _ denotes the actual number of people in the *i*-th test image, and *Z*_*i*_′ denotes the corresponding estimated count, i.e., the model output.

### 4.2. Ground Truth Generation

We follow the scheme used in previous studies [7, 8, 14] to prepare a ground truth density map. To ensure that the density map adapts to various conditions of crowd images, it can be expressed as *F*(*x*) with *N* heads. *F*(*x*) is obtained by convolving the delta function *δ*(*x* − *x*_*i*_) with a Gaussian kernel *G*_*σ*_*i*__(*x*) normalized to 1:

(6) $$\begin{array}{c} F\left(x\right)=\sum_{i=1}^{N}\delta \left(x-x_{i}\right)\times G_{\sigma_{i}}\left(x\right),\;\text{with }\sigma_{i}\;=\beta \bar{d^{i}}, \end{array}$$

where *x*_*i*_ represents per pedestrian head in a pixel, *σ*_*i*_ represents the crowd distribution of all the images in the dataset, *β* is a constant, and $\bar{d^{i}}$ represents the average distance of *k* nearest neighbors of the target. In our experiments, we follow a previously proposed configuration [8]. Certain parameters are set to fixed values (*β*=0.3 and *k*=3). The parameter settings for different datasets are listed in Table 1.

### 4.3. Training Details

MANet has an end-to-end structure. The training process is very simple. We set the training batch size to 1. MANet uses standard SGD with momentum (0.9) as the optimization method. Furthermore, we employ a random Gaussian initialization with a 0.01 standard deviation. The initial learning rate is set to 1*e* − 5. The learning rate decreases as the number of iterations increases.

### 4.4. Comparisons with State-of-the-Art (SOTA) Methods

We illustrate the result of our method using four challenging datasets. These datasets include different crowd situations, such as dense and sparse scenes. We present the density estimation results generated using MANet and discuss the problems in the model based on the results.

#### 4.4.1. ShanghaiTech Dataset

ShanghaiTech [7] has 1198 crowd images captured in sparse scenes. The images are divided into two datasets: Part_A and Part_B. Part_A comprises 300 training images and 182 testing images. Part_B comprises 400 training images and 316 testing images. The number of people in the image varies from 9 to 578.

**Figure 6:**
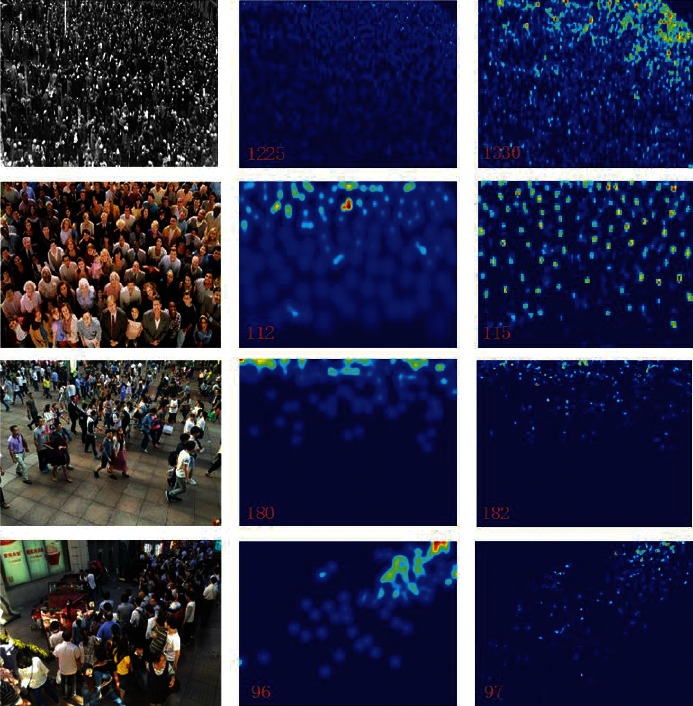
Comparison of visual results using the ShanghaiTech database. The first, second, and third columns contain test samples, the corresponding ground truth, and generated density map, respectively.

The test and visualization results obtained using the ShanghaiTech dataset are listed in Table 2 and illustrated in Figure 6. On Part_A, our approach outperforms PACNN [38], the most recently proposed method, by 1.49% and 10.2% in terms of MAE and MSE, respectively. These are good, although not exceptional results. Compared to the results obtained using PACNN [38], the results obtained using the proposed method on Part_B are not as good as those obtained on Part_A. This is because the image sources differ. The images in Part_A were downloaded randomly from the Internet, and the crowd density is very high. The images in Part_B were obtained in street scenes with a low crowd density and relatively complex backgrounds compared with images in Part_A. Our proposed network handles the multiscale problem well; however, it does not completely solve the problem of complex backgrounds. Many latest studies have added an attention mechanism, which improves the effect in some cases [14, 38].

#### 4.4.2. UCF_CC_50 Dataset

UCF_CC_50 [16] contains 50 crowd images with a total of 63974 people. The number of annotated people ranges from 94 to 4543 (an average of 1280). Fivefold cross-validation is the most commonly used method on this dataset.

**Figure 7:**
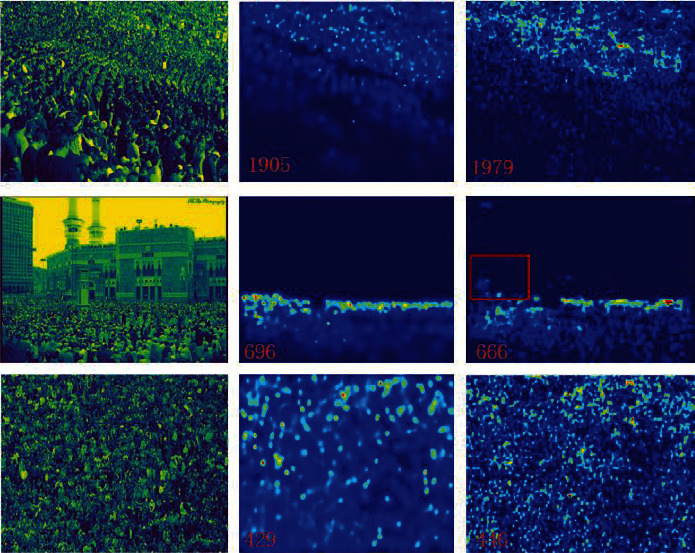
Comparison of visual results on UCF_CC_50. The first, second, and third columns show test samples, the corresponding ground truth, and the generated density maps, respectively. The box outlined in red represents an area where we mistook the background for a head.

The test and visualization results obtained using the UCF_CC_50 dataset are presented in Table 3 and illustrated in Figure 7. UCF_CC_50 is a very challenging dataset. It is a small dataset, and the resolution of the images is not high. The images are of pedestrians captured from different perspectives; therefore, scale variations are obvious. The MAE and MSE values obtained using the proposed method are 240.8 and 311.5, respectively; these values are 7.09% and 7.26% higher than those obtained using SPN [14]. Only some images in this dataset have background interference. These findings also prove that the proposed method achieves good results when handling small datasets with large-scale changes and dense crowds.

#### 4.4.3. WorldExpo'10 Dataset

WorldExpo'10 [15] includes images captured using 108 different surveillance cameras, containing 3,980 training frames in 1,132 video sequences, which can provide the cross scene to evaluate a model. The regions of interest are provided for all scenes.

**Figure 8:**
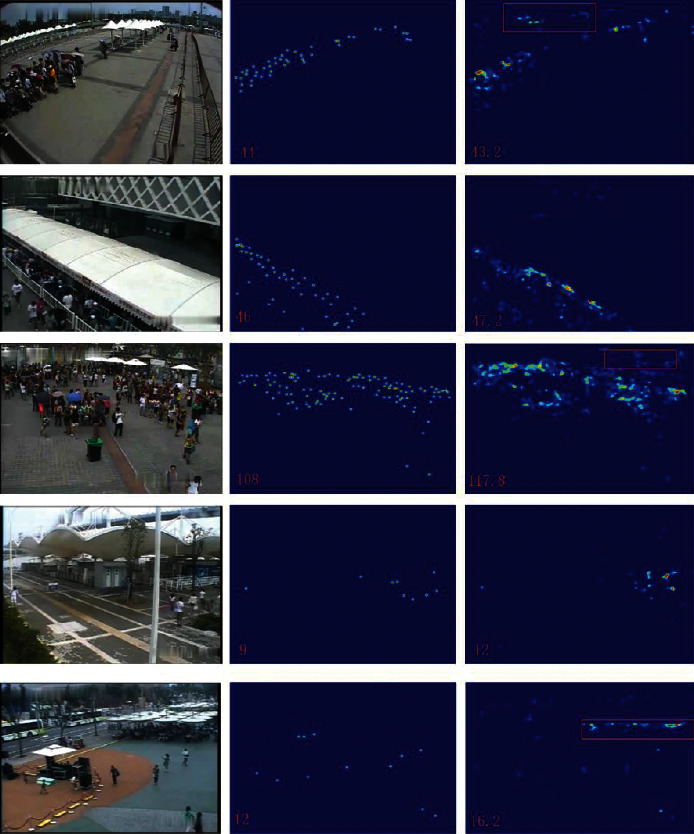
Comparison of visual results on WorldExpo'10. The first, second, and third columns show test samples, the corresponding ground truth, and the generated density map, respectively. The box outlined in red represents the area where we mistook the background for a head.

The test and visualization results obtained using the WorldExpo'10 dataset are provided in Table 4 and illustrated in Figure 8. The dataset is divided into five different scenes with different degrees of background interference. We tested each of them, and the average score is 7.86. The best results are obtained in *S*1 and *S*5, i.e., 2.1 and 3.0, respectively. However, our results are not as good as those obtained using SOTA in other scenes [12, 38]. Relative to other datasets, the shooting distance is long, the crowd does not show obvious multiscale changes, and the background interference is higher. In this case, our approach still shows good performance.

#### 4.4.4. SmartCity Dataset

SmartCity [9] contains 50 images. When collecting data, the shooting angle was high. The dataset includes ten scenes such as scenes of a sidewalk and a shopping mall. The images are divided into indoor and outdoor scenes and contain few pedestrians. The number of pedestrians ranges from 1 to 14 (an average of 7.4).

**Figure 9:**
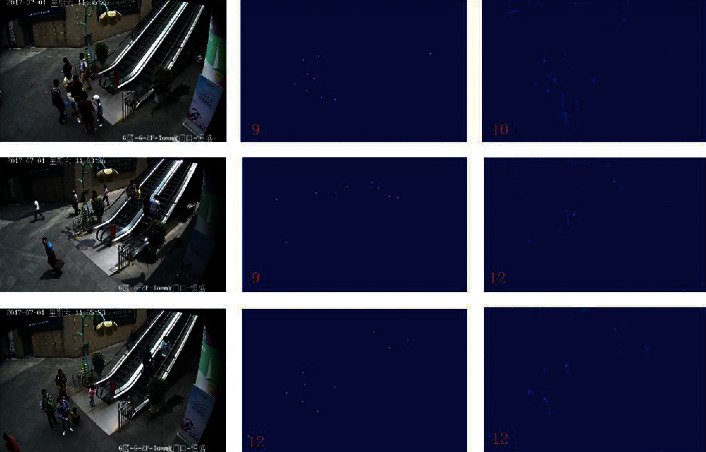
Comparison of visual results on Smart City. The first, second, and third columns contain test samples, the corresponding ground truth, and the generated density map, respectively.

The test and visualization results obtained using the SmartCity dataset are presented in Table 5 and illustrated in Figure 9. The MAE and MSE values are 8.2 and 9.6, respectively; these values are 4.65% and 17.24% higher than those obtained using SaCNN [9]. Differing from UCF_CC_50, the SmartCity dataset is small and the images have complex backgrounds, which are usually easy to identify. The results demonstrate that the proposed model shows good performance on small datasets with images of sparse crowds.

**Figure 10:**
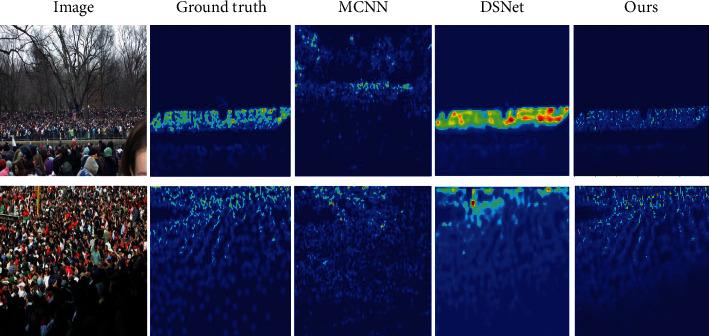
Comparison of the visualization results obtained using our method and those obtained using SOTA methods. From left to right, test samples, ground truth, and visualization results were obtained using MCNN [7], DSNet [41], and MANet.

As shown in the table, our method obtains the lowest MAE and MSE values on multiple datasets. These results demonstrate the effectiveness of the proposed method, particularly in the case of a high-density crowd in an image. This observation not only proves the effectiveness of our method but also demonstrates its robustness. We compare the visualization results of the proposed method with those obtained using SOTA methods. The density map produced by our model is of higher quality and closer to the original map (Figure 10). This also proves that our model can retain more multiscale and contextual information. However, our results also indicate that the proposed model has some limitations. Occasionally, objects in the background of an image are mistakenly classified as pedestrians in a crowd. These phenomena are indicated by boxes outlined in red (Figures 7 and 8). This type of problem may lead to other problems with our model under the background of similar goals, and we must address this issue through certain mechanisms.

### 4.5. Ablation Experiments

In this section, we describe several ablation studies, including the CSPN and dense connection operations, to demonstrate the effects of different modules in our proposed MANet.

#### 4.5.1. Effectiveness of CSPN

To prove the effectiveness of the CSPN structure, we conduct multiple ablation experiments. (1) The last convolution layer in the MCNN is replaced with the CSPN (MCNN + CSPN). (2) The last convolution layer in the MCNN network is replaced with the SAN of SANet (MCNN + SAN). (3) The backend of CSRNet is replaced with the CSPN (CSRNet + CSPN). (4) The CSPN in MANet is replaced by an ordinary convolution (CNet) (Table 6).

Our experiment on MCNN proves that the CSPN is effective. The MSE of MCNN is reduced from 110.2 to 92.4, and the MSE is reduced from 173.2 to 157.5. However, our effect is similar to that of SAN. Compared with CSRNet, the results are similar. Moreover, the self-ablation experiment proved its effectiveness.

#### 4.5.2. Effectiveness of Dense Connections

To clarify the contributions of our proposed dense connections, the following two architectural configurations are evaluated: (1) the structure with added dense connections is called MANet-1 and (2) the structure without added dense connections is called MANet-2. The final results are shown in Table 7.

The results demonstrate that the incorporation of dense connections provides better results than not including the connections. More connections help the model retain features; however, the disadvantage is that a large number of features require more computational resources and training time.

## 5. Conclusion

In this study, we proposed MANet, an innovative encoder-decoder structure for crowd counting. MANet comprises a FEE and a DMD. FEE uses dense connections to integrate the features extracted from the CSPN, a multiscale aggregation network, to obtain multiscale and contextual information. The DMD adopts deconvolutions and fusion operations to obtain features containing detailed information to realize high-quality density maps. We conducted numerous experiments on the model using the four datasets. Experimental results show that the proposed MANet performs well on MAE and MSE. The focus of future work will be on increasing the attention mechanism for an improved distinction between crowds and backgrounds.

## Figures and Tables

**Table 1:** Parameter settings for different datasets.

Datasets	Parameter settings
ShanghaiTech part_A	*σ*_*i*_=4
ShanghaiTech part_B	*σ*_*i*_=15
WorldExpo'10	*σ*_*i*_=3
UCF_CC_50	Geometry-adaptive kernels
SmartCity	*σ*_*i*_=15

**Table 2:** MAE and MSE results using various methods (ShanghaiTech dataset).

Methods	Part_A		Part_B	
	MAE	MSE	MAE	MSE
Zhang et al. [15]	181.8	277.7	32.0	49.8
MCNN [7]	110.2	173.2	26.4	41.3
CP-CNN [6]	73.6	106.4	20.1	30.1
CSRNet [8]	68.2	115.0	10.6	16.0
SANet [10]	67.0	104.5	8.4	13.6
SPN [14]	61.7	99.5	9.4	14.4
PACNN [38]	66.3	106.4	8.9	13.5
MANet	65.31	**95.54**	10.2	16.5

**Table 3:** MAE and MSE results using various methods (UCF_CC_50 dataset).

Methods	MAE	MSE
Zhang et al. [15]	467.0	498.5
MCNN [7]	377.6	509.1
Switch-CNN [4]	318.1	439.2
CP-CNN [6]	295.8	320.9
CSRNet [8]	266.1	397.5
SANet [10]	258.4	344.9
SPN [14]	259.2	335.9
MANet	**240.8**	**311.5**

**Table 4:** MAE and MSE results using various methods (WorldExpo'10 dataset).

Methods	S1	S2	S3	S4	S5	Avg.
Zhang et al. [15]	9.8	14.1	14.3	22.2	3.7	12.9
MCNN [11]	3.4	20.6	12.9	13.0	8.1	11.6
Switch-CNN [7]	4.4	15.7	10.0	11.0	5.9	9.4
CP-CNN [10]	2.9	14.7	10.5	10.4	5.8	8.9
CSRNet [12]	2.9	11.5	8.6	16.6	3.4	8.6
PACNN [38]	2.3	12.5	9.1	11.2	3.8	7.8
MANet	**2.1**	13.5	9.3	11.4	**3.0**	7.86

**Table 5:** MAE and MSE results using various methods (Smart City dataset).

Methods	MAE	MSE
Zhang et al. [15]	40.0	46.2
Sam et al. [4]	23.4	25.2
SaCNN [9]	8.6	11.6
MANet	**8.2**	**9.6**

**Table 6:** CSPN validation results.

Methods	ShanghaiTech Part_A	
Evaluation	MAE	MSE
MCNN [7]	110.2	173.2
MCNN + CSPN	92.4	157.5
MCNN + SAN	95.3	155.7
CSRNet [8]	68.2	115.0
CSRNet + CSPN	66.5	108.6
CNet	96.7	132.3
MANet	65.31	95.54

**Table 7:** Comparison of different structures using a benchmark dataset.

Methods	ShanghaiTech part_A	
Evaluation	MAE	MSE
MANet-1	87.31	108.89
MANet-2	65.31	95.54

## Data Availability

The data used to support the findings of this study are available from the corresponding author upon request.
